# Evaluation of Nutrition Risk Screening Score 2002 (NRS) assessment in hospitalized chronic kidney disease patient

**DOI:** 10.1371/journal.pone.0211200

**Published:** 2019-01-24

**Authors:** Martin Müller, Suzan Dahdal, Mo Saffarini, Dominik Uehlinger, Spyridon Arampatzis

**Affiliations:** 1 Department of Emergency Medicine, Inselspital, Bern University Hospital, University of Bern, Bern, Switzerland; 2 Department of Nephrology, Hypertension and Clinical Pharmacology, Inselspital, Bern University Hospital, University of Bern, Bern, Switzerland; 3 ReSurg SA, Chemin de Vuarpilliere 35, Nyon, Switzerland; University of Mississippi Medical Center, UNITED STATES

## Abstract

**Background:**

Although chronic kidney disease (CKD) patients are particularly prone to malnutrition, systematic nutritional screening is rarely routinely performed during hospitalization. The primary aim of this study was to determine the prevalence of malnutrition (as captured by the nutritional screening score NRS) in hospitalized CKD patients and explore the impact of malnutrition on hospital mortality.

**Methods:**

All patients admitted to the tertiary nephrology department of the University hospital of Bern Inselspital over a period of 12 months were included in this observational study. The risk for malnutrition was assessed within 24h of admission by the NRS. Demographic, clinical, and outcome data were extracted from the patient database. The primary outcome was in-hospital mortality. The secondary outcomes were length of hospitalization and hospitalization costs. Multilevel mixed-effect logistic regression model analysis was performed to determine the association of in-hospital mortality and risk of malnutrition (NRS score≥3).

**Results:**

We included 696 eligible hospitalizations of 489 CKD patients. Hospitalized patients had a median age of 64 years (interquartile range (IQR), 52–72), 35.6% were at risk of malnutrition (NRS≥3). After adjustment for the identified confounders (Case weight, Barthel index, and CKD stage) multivariate analysis confirmed an independent and significant association between higher in-hospital mortality with NRS≥3 [OR 2.92 (95% CI: 1.33–6.39), P<0.001]. Furthermore, in multivariate analysis the risk of malnutrition was associated with longer length of hospitalization [Geometric mean ratio: 1.8 (95% CI: 1.5–2.0), p<0.001] and with increased hospitalization costs [Geometric mean ratio: 1.7 (95% CI: 1.5–1.9), p<0.001]).

**Conclusions:**

Malnutrition in CKD patients, as captured by NRS>3, is highly prevalent among hospitalized CKD patient and associated with prolonged hospital stay and increased in-hospital mortality.

## Introduction

The prevalence of malnutrition in chronic patients is substantial and varies significantly depending on the screening instruments used for assessment. In hospitalized patients, malnutrition is observed in 20–60% and is associated with increased morbidity, mortality, and healthcare costs [1–5]. Chronic kidney disease (CKD) patients are commonly depleted of protein and energy stores and particularly prone to develop malnutrition [6, 7]. Yet, many CKD patients with a high risk of malnutrition remain undetected during hospitalization, due to the lack of standardized nutritional screening tools. Several studies have found associations between nutritional risk and adverse clinical outcome using various screening tools such as the Subjective Global Assessment (SGA) [8], the Malnutrition Screening Tool (MST) and the Nutrition Risk Screening 2002 (NRS) [9]. So far, the most successfully evaluated screening tools in CKD patients are the SGA or one of its adaptations [10, 11]. The NRS combines both a measure of current potential undernutrition and a measure of disease severity and has been validated in various patient groups. It was shown to be reliable to identify hospital patients at risk of malnutrition and was therefore recommended by the European Society of Clinical Nutrition and Metabolism (ESPEN) [12]. The NRS has equivalent sensitivity and specificity compared to the SGA, but is quicker and simpler to use, and thus requires considerably less examiner training [13, 14]. There is scant information regarding the standardized use of the NRS in tertiary nephrology wards. If malnutrition imparts added functional impairment in patients with CKD, there might be a strong rationale for screening and treating malnutrition during hospitalization in the hopes of improving functional status and outcomes.

Thus, the primary aim of this study was to evaluate the prevalence of malnutrition risk among hospitalised CKD patients by the standardised implementation of the nutritional risk screening score NRS and to examine the impact of malnutrition, on relevant clinically endpoints, i.e. hospital mortality, length of hospitalization, and hospitalization costs.

## Materials and methods

### Study design and patients

In this quality control study, the nursing staff assessed the nutritional status of all adult patients (>18 years old) hospitalized for more than 24 hours in the nephrology department of the Inselspital, University Hospital of Bern, during a 12-month period. The assessment was based on the NRS questionnaire [9].

### Exposure: Risk of malnutrition

The risk for malnutrition was defined as an NRS-2002 score of ≥3 points [15]. The NRS was performed in a bedside patient interview.

### Primary outcome: In-hospital mortality

The primary outcome was in-hospital mortality coded as a binary variable. The strength of association between the risk of malnutrition and in-hospital mortality was quantified.

The binary outcome in-hospital mortality in combination with logistic regression analysis was preferred over survival analysis with Cox regression as i) additional assumptions (proportional hazard assumptions) did not need to be tested, ii) there were no censored patients, and iii) the time to event (death) was not of particular interest in this study.

### Secondary outcomes: Length of hospital stay and costs

The secondary outcomes were length of hospital stay in days from admission until hospital discharge (including in-hospital death) and hospitalization costs (in Swiss Francs) obtained from the hospital electronic system.

### Data collection: Potential confounders and others

Patients with the risk of malnutrition appear to have more comorbidities and to be more elderly. The association between risk of malnutrition and the outcomes therefore needed to be controlled for some clinical parameters and comorbidities.

We obtained different potential confounders as surrogate parameters for functional status and acute and chronic morbidity.:

First, the Barthel index was used to measure activities of daily living (ADLs) and was completed based on structured interviews [16]. A Barthel index cutoff of <80 points was defined as indicating dependence on others [16].

Second, we collected pertinent clinical information, including sex and age. Reasons for hospitalization were routinely gathered from the hospital electronic medical system for coding of diagnosis-related group (DRG) codes to further characterize the patients.

Third, routine laboratory data, when available, were extracted when available from charts or from the electronic laboratory database of the hospital as surrogate parameters for acute or chronic illness. The following laboratory variables were tabulated: haemoglobin, leucocyte count, sodium, potassium, calcium, creatinine, albumin, and CRP.

Fourth, nephrology-specific variables such as previous renal transplantation, the grade of chronic kidney disease (CKD grade: I mild–V chronic kidney failure) and acute kidney disease (AKIN grade: I risk–III acute kidney failure) that are routinely stored in the medical records were obtained.

Fifth, we used the case weight to further control for comorbidity. One positive integer value, the so-called case weight, is assigned to every hospitalization on the basis of the diagnosis-related group and the comorbidities of the patient for administrative purposes. The case weight is often between 0.2 to 5 at our nephrological ward; the higher the value, the more complex and morbid the patient.

For the purpose of this study, we used the case mix as a surrogate marker for comorbidity in combination with the Barthel index, sociodemographic data, nephrological conditions, and the laboratory values.

### Ethical considerations

This was an observational quality control study and all data were anonymized prior to analysis. The study was approved by the Cantonal Ethics committee and was in accordance with Helsinki Declaration of Human Rights.

### Statistical analysis

Statistical analyses were performed using Stata 13.1 (StataCorp, The College Station, Texas, USA). Data are presented as percentages for categorical data or as medians with interquartile range (IQR) as most of the continuous variables were not normally distributed. Comparisons between categorical variables were performed by Fisher’s exact test. Differences in continuous variables between two groups were assessed using the Mann-Whitney’s U tests.

To incorporate the impact of multiple hospitalizations of the same patient, a multilevel mixed-effects logistic regression model (*–melogit–*command) for the association of the risk of malnutrition and in-hospital mortality as well as a multilevel mixed-effects linear regression (*–mixed–*command) with the patient identification number as the random effect was used to quantify the strength of association of the risk of malnutrition with the length of hospitalization respectively with hospitalization costs. The strength of the association between risk of malnutrition and in-hospital mortality was quantified through the obtained odds ratio (OR, with 95% confidence interval). The variables length of hospitalization and hospitalization costs were ln-transformed before analysis as it was strongly skewed, the obtained regression coefficients were exponentiated. Thus, the presented exponentiated regression coefficients correspond to the geometric mean ratio of the non-log transformed values of length of hospitalization and hospitalization costs [17].

After univariate analysis, all potential confounders for multivariable modelling were identified in univariate through a p-value <0.2 of the association with the exposure risk of malnutrition and the variable. The final model for both the primary and secondary outcome was determined through stepwise exclusion of variables with a p-value of p>0.1 after adjustment, starting with the variable with the highest p-value.

For sensitivity analysis a logistic and linear regression i) without a random effect and ii) with restriction to the first hospitalization of a patient adjusted for the identified confounders was performed and presented.

Statistical differences were considered significant when p<0.05.

## Results

**Fig 1:**
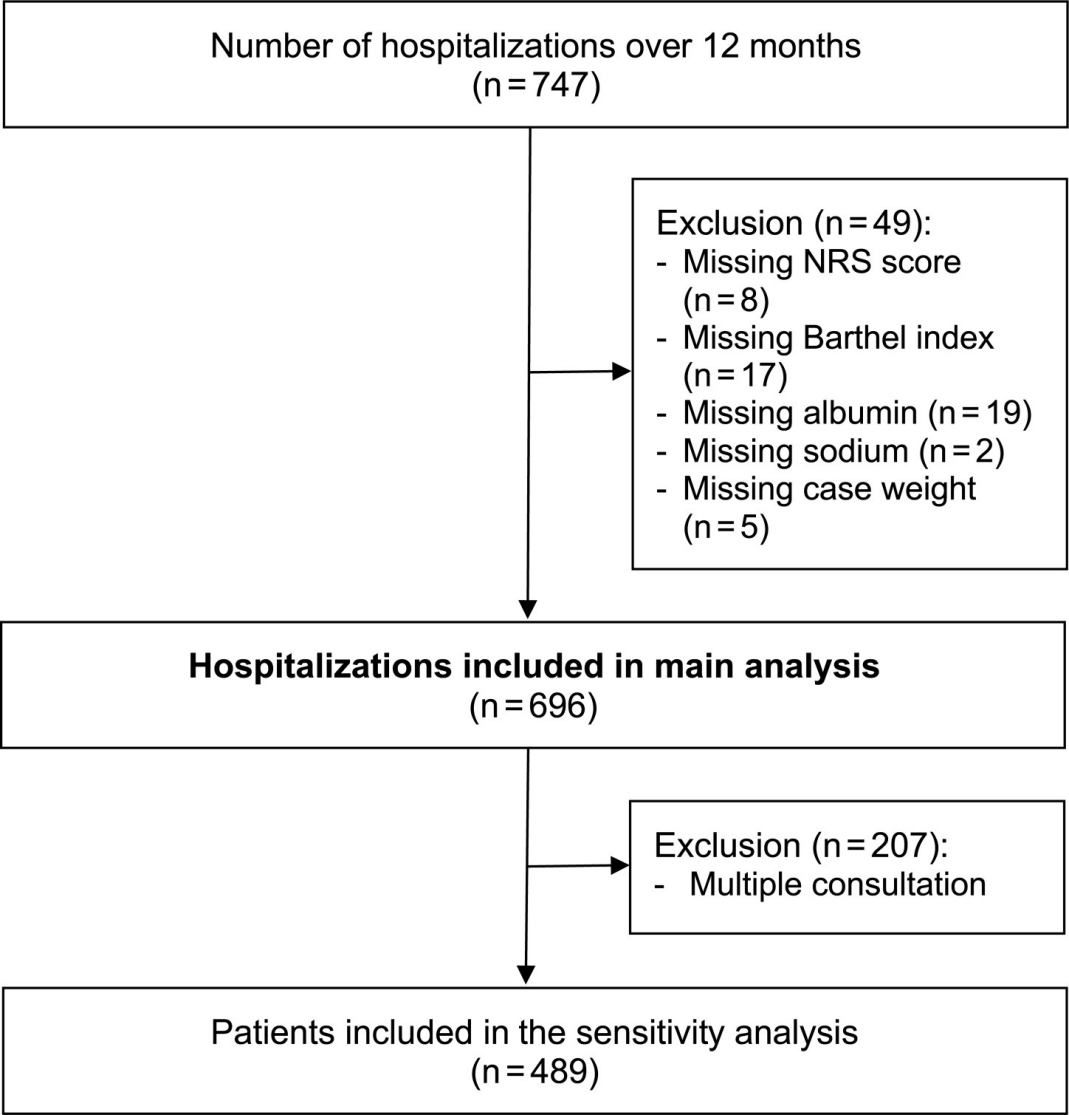
Flowchart. Abbreviations: NRS, Nutrition Risk Screening.

Demographic and hospitalization characteristics are shown in Table 1. A total of 747 hospitalizations of 508 patients were admitted to the nephrology department during the 12-month study period. In total, 6.8% (n = 51) had to be excluded because of incomplete documentation (Fig 1). Thus, 696 hospitalizations of 489 patients were included in the main analysis.

**Table 1:** Demographic and hospitalization characteristics of the included 696 (100%) hospitalizations. Abbreviations: DRG, diagnosis related group.

Number of patients, n (%)	489 (70.3)
At risk of malnutrition i.e. NRS≥3, n (%)	248 (35.6)
Gender	
Male, n (%)	421 (60.5)
Female, n (%)	275 (39.5)
Age (years; median and interquartile range in brackets)	64 (52–72)
Reason of hospitalization due to group related diagnosis (DRG), n (%)	
Kidney and urinary tract including transplantation related problems	233 (33.5)
Circulatory problems	115 (16.5)
Musculoskeletal system and connective tissue diseases	72 (10.3)
Digestive system disorders	46 (6.6)
Infectious diseases	44 (6.3)
Endocrine, nutritional and metabolic system diseases	27 (3.9)
Respiratory system diseases	27 (3.9)
Blood and blood forming organs and immunological disorders	19 (2.7)
Ear, nose, mouth and throat diseases	16 (2.3)
Nervous system disorders	15 (2.2)
Others	82 (11.8)
Number of admissions per patient, n (%)	
1 admission	373 (76.3)
2 admissions	66 (13.5)
3 admissions	29 (5.9)
≥4 admissions	21 (4.3)

Based on the NRS score from all hospitalized cases, 248 admissions (35.6%) were at risk of malnutrition (score ≥ 3), see Table 1. Most of the admitted patients were male (60%), with a mean age of 64 years (IQR 52–72) and presented mostly as emergency admissions (59%). The main reason for hospitalization based on the primary group related diagnosis (DRG) were diseases of the kidney, urinary tract and transplantation related problems (34%). Almost half of the patients had at least one rehospitalization during the study period generating a total of 747 admissions.

The comparison of conditions related to hospitalization between patients with NRS score<3 and those with NRS score≥3 are shown in Table 2.

**Table 2:** Comparison of hospitalization conditions and characteristics between hospitalizations with NRS score <3 and those with NRS score≥3.

	NRS score<3 (n = 448)		NRS score≥3 (n = 248)		Total (n = 696)		P-value
Age, med (IQR)	61.0	(48–69)	70.0	(60–77)	64.0	(51.5–72)	<0.001
Sex, n (%)							
Male	276	(61.6)	145	(58.5)	421	(60.5)	
Female	172	(38.4)	103	(41.5)	275	(39.5)	0.417
CKD grade, n (%)							
I	40	(8.9)	10	(4.0)	50	(7.2)	
II	47	(10.5)	11	(4.4)	58	(8.3)	
IIIa	45	(10.0)	14	(5.6)	59	(8.5)	
IIIb	74	(16.5)	41	(16.5)	115	(16.5)	
IV	108	(24.1)	74	(29.8)	182	(26.1)	
V	134	(29.9)	98	(39.5)	232	(33.3)	<0.001
AKIN grade, med (IQR)	0	(0–0)	1	(0–3)	0	(0–1)	<0.001
Renal transplantation, n (%)	94	(21.0)	23	(9.7)	118	(17.0)	<0.001
Case weight, med (IQR)	0.8	(0.5–1.5)	1.7	(1–2.9)	1.0	(0.6–2.1)	<0.001
Barthel index, med (IQR)	80.0	(55–100)	50.0	(25–77.5)	70.0	(40–95)	<0.001
**Laboratory data, [med (IQR)]**							
Sodium, mmol/L	138.0	(135–140)	136.0	(133–139)	137.0	(134–140)	<0.001
Potassium, mmol/L	4.3	(3.9–5.0)	4.5	(4–5.2)	4.4	(3.9–5.1)	0.016
Calcium, mmol/L	2.2	(2.1–2.4)	2.2	(2.1–2.3)	2.2	(2.1–2.3)	0.268
Creatinine, μmol/L	216	(129–388)	277	(166–443)	236	(138–402)	<0.001
CRP, mg/L	13	(4–49)	28	(8–81)	18.0	(5–60)	<0.001
Albumin, g/L	32	(23–37)	28	(24–35)	30.0	(23–36)	0.088
Haemoglobin, g/L	110.0	(96–125)	101.5	(89–116.5)	107.0	(93–124)	<0.001
Leucocytes, G/L	7.6	(5.7–11.1)	8.0	(5.8–11.2)	7.8	(5.8–11.1)	0.398
**Administrative data, med (IQR)**							
Costs, Sfr,	9,180	(5,513–18,5723)	25,721	(14,387–53,110)	13,268	(6,868–27,694)	<0.001
Length of stay	4.0	(2–7)	11.0	(5.5–21)	5.0	(2–12)	<0.001
In-hospital mortality, n (%)	10	(2.2)	27	(10.9)	37	(5.3)	<0.001

Abbreviations: AKIN, acute kidney injury; CKD, chronic kidney disease; CRP, C reactive protein; IQR, interquartile range; med, median; NRS, nutrition risk screening; Sfr, Swiss Francs.

Patients at risk of malnutrition (NRS≥3) were older, had a higher case index weight and a lower Barthel index than patients without the risk of malnutrition (NRS<3). Based on the biochemical profile at admission serum sodium, potassium, albumin, CRP levels and haemoglobin were lower in patients at malnutrition risk compared to patients with no risk for malnutrition (p<0.2).

Furthermore, patients with NRS score≥3 generated higher costs, had a longer hospitalization stay and higher in-hospital mortality (10.9% vs. 2.2%) compared to patients with NRS score <3, all p<0.001.

### Univariate and multivariate analysis of possible nutritional risk factors concerning in-hospital mortality

In univariate analysis, in-hospital mortality was significantly associated with risk of malnutrition (NRS score ≥3) with an odds ratio of 5.4 (95% CI: 2.5–11.3, p<0.001), see Table 3. Furthermore, malnutrition was associated with a longer length of hospital stay and increased hospitalization costs. The geometric means for length of hospital stay and for hospitalization costs in patients with risk of malnutrition was 2.8 (95% CI: 2.4–3.3, p<0.001) and 2.6 (95% CI: 2.2–2.9, p<0.001) times higher compared to patients without risk of malnutrition.

**Table 3:** The univariate association of the risk of malnutrition (NRS score≥3) and the primary outcome in-hospital mortality and secondary outcomes length of hospital stay and hospitalization costs (ln-transformed). Abbreviations: NRS, nutrition risk screening.

In-hospital mortality	Odds ratio (95% CI)		p
Risk of malnutrition (NRS score≥3)	5.35	(2.54–11.25)	<0.001
**Length of stay (ln-transformed)**	**Geometric mean ratio (95% CI)**		
Risk of malnutrition (NRS score≥3)	2.83	(2.44–3.30)	<0.001
**Hospitalization costs (ln-transformed)**	**Geometric mean ratio (95% CI)**		
Risk of malnutrition (NRS score≥3)	2.55	(2.23–2.93)	<0.001

All Variables shown in Table 2 with at least a p-value of <0.2 were considered as potential confounders and were controlled for in the multivariate analysis. As creatinine is already reflected in the variables CKD and AKIN it was not additionally considered in the final model. The Barthel index was considered as a binary parameter (<80 vs. ≥80).

Stepwise, variables with a p-value >0.1 were removed to obtain the final model for both the primary and secondary outcomes.

The final multivariate analysis between in-hospital mortality as well as length of hospitalization and hospitalization costs with the risk of malnutrition (NRS score ≥3) are shown in Table 4.

**Table 4:** Multivariate analysis between in-hospital mortality and a NRS score<3 and those with NRS score≥3 controlled for identified factors associated with a NRS score<3 and in-hospital mortality. Abbreviations: AKIN, acute kidney injury; CKD, chronic kidney disease; CRP, C reactive protein; NRS, nutrition risk screening.

In-hospital mortality	Odds ratio (95% CI)*		p
Risk of malnutrition (NRS score≥3)	2.92	(1.33–6.39)	0.008
**Length of stay (ln-transformed)**	**Geometric mean ratio (95% CI)**^+^		
Risk of malnutrition (NRS score≥3)	1.75	(1.51–2.03)	<0.001
**Hospitalization costs (ln-transformed)**	**Geometric mean ratio (95% CI)**^#^		
Risk of malnutrition (NRS score≥3)	1.66	(1.47–1.88)	<0.001

*adjusted for CKD grade, case weight, Barthel index (≥80).

^+^adjusted for AKIN grade, case weight, Barthel index (≥80), potassium, CRP, albumin, haemoglobin, leucocytes.

^#^adjusted for AKIN grade, case weight, Barthel index (≥80), CRP, albumin, haemoglobin, leucocytes.

In multivariate analysis controlled for the parameter CKD grade, case weight and Barthel index (≥80), the risk of malnutrition (NRS score ≥ 3) was significantly associated with in-hospital mortality with an odds ratio of 2.9 (95% CI: 1.3–6.4, p = 0.008).

Furthermore, the risk of malnutrition was associated with longer length of hospitalization (Geometric mean ratio: 1.8, 95% CI: 1.5–2.0, p<0.001) and with increased hospitalization costs (Geometric mean ratio: 1.7, 95% CI: 1.5–1.9, p<0.001). The associations were adjusted for AKIN grade, case weight, Barthel index and the laboratory values CRP, albumin, haemoglobin and leucocytes as well in the case of the association with length of hospitalization potassium (Table 4).

### Sensitivity analysis for the primary outcome

Using the same identified confounder (CKD grade, case weight and Barthel Index) in a logistic regression without the random effect for rehospitalization of a patient did not change the odds ratio between the risk of malnutrition and in-hospital mortality (2.9, 95% CI: 1.3, 6.4). After restriction of the analysis to the first hospitalization of a patient (n = 489), controlling for the same confounder, the odds ratio between risk of malnutrition and in-hospital mortality increased (OR 4.1, 95% CI: 1.3, 13.2, p = 0.019).

## Discussion

Patients with CKD are particularly vulnerable to the deleterious effects of malnutrition although malnutrition often remains unrecognized [6]. The primary aim of the study was to explore the prevalence of malnutrition among patients admitted on a tertiary nephrology ward as captured by the NRS screening tool. This observational study shows that more than one-third of hospitalised CKD patients were at risk of malnutrition. Furthermore, such patients have significantly higher in-hospital mortality, generate higher healthcare costs, and have a longer length of hospitalization.

Our results are in accordance with previous studies performed mainly in internal medicine wards showing that a considerable proportion of hospitalised patients were classified as malnourished (NRS ≥ 3) [18, 19]. Similar in a recent study among hospitalized CKD patients, malnutrition was also highly prevalent (40%) and it affected the length of hospitalization, although mortality was not explored [20]. Several studies suggested that awareness of nutritional status and treatment of malnutrition are often insufficient and that the challenge lies in implementing appropriate screening tools leading to proper nutritional evaluation and support [21–24]. Our findings highlight the importance of systematic screening upon admission in hospital wards.

The EuroOOPS, a large multicentre multinational study showed that malnutrition in general hospital patients, as defined by the NRS, is associated with significantly higher mortality, complication rate and length of hospitalization [25]. Analogous associations were recently confirmed in another large single centre study in Switzerland [26], where in contrast to the EuroOOPS, surgical and intensive care patients were excluded. The impact of malnutrition on mortality and length of hospitalization was also demonstrated in our study. The median duration of hospitalization of the undernourished patients was almost twice as long as of the subjects with normal nutritional status. Moreover, the results of multiple regression analysis showed that malnutrition was an independent factor for the prolongation of hospitalization. The costs of treating disease-related malnutrition in Europe are estimated around € 170 billion annually [27]. Our analysis revealed that the costs of treating CKD patients at risk of malnutrition are nearly triple those of treating CKD patients that are not at risk

It is important to note that restrictions in renal diets often contradict normal nutritional recommendation, and even if limiting intake of sodium, potassium, phosphates, and fluids can prevent complications, problems occur if such restrictions are not accompanied with counselling on alternative dietary choices and strategies to maintain adequate nutrition [28]. There is very little evidence regarding the benefits of nutritional interventions in CKD patients, though ongoing multi-centric studies could provide further evidence to support such treatment strategies.

It is well known that patient on ICU are at high risk for malnutrition. Malnutrition is related to adverse outcome and different interventional studies were performed to find optimal nutrition strategies for such patients [29]. Patients with kidney failure in ICU are more and more recognized as especially vulnerable for malnutrition and are at higher risk for 1-year mortality [30]. Our study highlights the importance to recognize kidney failure in patients not only in ICU but also in normal wards as risk factor for malnutrition.

### Implication of our study

Currently ESPEN recommends the use of NRS in hospitalized patients but there is neither a universally accepted screening tool nor a diagnostic biochemical marker for identifying malnutrition risk in CKD patients. An easy-to-apply, reliable, and valid screening tools as the NRS score can prompt diagnosis of malnutrition induce appropriate nutrition interventions and decrease adverse outcomes and healthcare costs. The challenge lies more in implementing appropriate screening tools such as the NRS and proper nutritional support in randomized controlled trials in order to evaluate the impact of nutritional strategies in CKD patients, which are at substantial nutritional risk.

### Strengths and weaknesses

This study has some limitations. Our results are based on the admission NRS score with no longitudinal follow-up. Also factors like past medical history and comorbidities like diabetes and high blood pressures were not addressed although we used surrogate parameter for morbidities such as case-mix index and Barthel index. We did not compare NRS to other nutritional status assessment tools or implemented any appetite questionnaires. Despite the single-centre nature of our study, the main strength lies in the representative CKD patient sample within a prospective observational setting.

### Conclusion

In conclusion, pre-existing malnutrition is substantial among CKD patients, associated with increased resource use, prolonged hospital stays and in-hospital mortality. Since patient with CKD represent a high-risk population for malnutrition-associated adverse outcomes, routine evaluation of nutritional status at hospital admission should become a standardized procedure.

## Supporting information

S1 File Dataset of the study. (XLS)
